# Characteristics Associated with Disclosure Status to Sexual Partners among Kenyan Women

**DOI:** 10.16966/2380-5536.166

**Published:** 2019-09-06

**Authors:** Haley Greene, Pauline E Jolly, Tierra Johnson, Ruby Galarza, Walter Jaoko, Luz A Padilla

**Affiliations:** 1 Department of Epidemiology, Ryals School of Public Health, University of Alabama at Birmingham, Alabama, USA; 2 Department of Medical Microbiology, University of Nairobi, Nairobi, Kenya

**Keywords:** HIV/AIDS, HIV Disclosure, Women, Sexual Risk Behaviors, Kenya

## Abstract

**Background:**

Kenya has the fourth-largest HIV epidemic across the globe. Disclosure of HIV-positive status plays a critical role in the prevention of HIV transmission. Disclosure, specifically to sexual partners, has been shown to foster safer sexual behaviors in addition to emotional, social, or monetary support from partners.

**Objective:**

This study was conducted to identify factors associated with HIV-positive status disclosure to sexual partners compared to disclosure to other than sexual partners.

**Methods:**

A cross-sectional study was conducted from May to August 2012 among 497 HIV-positive women 19–49 years of age who had sex in the past six months. Participants were recruited from the Kenyatta National Hospital and Mbagathi Direct Hospital in Nairobi, Kenya. A questionnaire was administered to obtain data on HIV disclosure. Bivariate and multivariable logistic regression analyses were conducted to identify factors associated with disclosure of HIV-positive status to sexual partners.

**Results:**

Of the 497 women, 349 reported to whom they had disclosed their HIV status. Approximately 34% had disclosed their HIV-positive status to a sexual partner. Women who disclosed to their sexual partners were 11 times more likely to be married or in a common-law relationship and 4 times more likely for their sexual partner or spouse to be the heads of the households. Frequency of condom use, belief that HIV disclosure is important for HIV prevention and control, knowledge of partner’s HIV status, and number of sex partners were also significantly associated with disclosing to a sexual partner.

**Conclusion:**

This study found a low rate of disclosure of HIV-positive status by women to sexual partners and identified a number of factors associated with disclosure to sexual partners. These findings can be used in designing interventions that focus on individuals who have not disclosed their HIV-positive status to their sexual partners by demonstrating the importance of disclosure and safe sex practices.

## Introduction

People living with HIV/AIDS (PLWHA) experience various barriers that may influence their decision to disclose their HIV status; for example, loss of social support, HIV-related discrimination, wish to protect oneself or others emotionally, concern about insurance or loss of employment, access to care and support services, or intimate partner violence [1,2]. The disclosure of HIV-positive status plays a critical role in the prevention of HIV transmission to others and the alleviation of its impact on quality of life, stigma, treatment understanding, and social support [2]. In order to manage the obstacles associated with HIV infection, disclosure of HIV-positive status specifically to sexual partners has been shown to create a relationship that fosters safer sexual behaviors in developing countries where women tend to be more dependent on their partners [3–5]. Disclosing one’s HIV-positive status is also related to the prevention of HIV transmission as well as emotional, social, or monetary support from partners [3–5]. While the benefits of HIV disclosure are thought to outweigh the risks, HIV disclosure status remains low in many African countries, and stigma and lack of social support are often associated with nondisclosure [6].

The average rate of HIV disclosure in the developing world is notably less than the average rate reported from studies in industrialized countries with percentages between 42% to 100% [3,4,7–9]. Rates of HIV disclosure to sexual partners vary significantly with percentages between 16.7% to 86% of women choosing not to disclose their HIV-positive status to their sexual partners in Africa [3,4,7–9]. In southern Africa, approximately 42% of PLWHA have disclosed their HIV-positive status to a sexual partner, and roughly, 72% of those who have not disclosed have multiple partners [8]. Additionally, chiefly attributed to barriers to disclosure such as separation or loss of sole source of income, women in Uganda were most likely to disclose their HIV-positive status to their sisters (21%) while men were most likely to disclose to their sexual partners (27%); in fact, only 22% of Tanzanian women have disclosed their HIV-positive status to their sexual partner [7,10].

Along with Mozambique and Uganda, Kenya holds the fourth-largest HIV epidemic across the globe [11]. The overall HIV prevalence was estimated at approximately 4.9% in 2017 with roughly 28,200 people dying from AIDS-related causes in Kenya that year [11]. The epidemic affects the general population and leaves groups of women who are African American, transgender, injection drug users, sex workers, or those with a low socioeconomic status more vulnerable to infection [11,12]. Compared to their male counterparts, young women are almost twice as likely to be infected with HIV in addition to having a higher prevalence of HIV when compared to men (5.2% *vs*. 4.5%) [11,12]. Research shows that women are less likely to disclose their HIV-positive status to their spouses, and the failure to disclose HIV-positive status to a sexual partner may influence the rates of HIV infection among the population [10]. This study sought to identify factors associated with HIV status disclosure by women to their sexual partners compared to women who disclosed to someone other than their sex partner.

## Materials and Methods

### Study sites and participants

**Figure 1:**
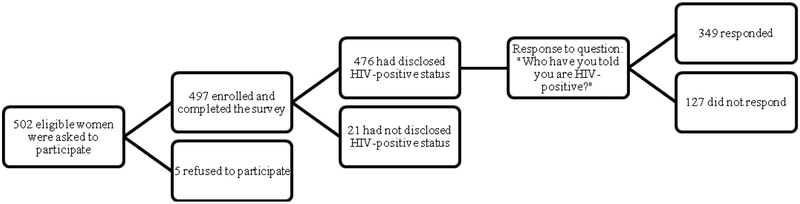
Flowchart showing recruitment of participants.

In 2012, 497 clinically confirmed HIV-positive women receiving care in Nairobi, Kenya at Kenyatta National Hospital (KNH) or Mbagathi District Hospital (MDH) participated in this cross-sectional study. Kenyatta National Hospital is the largest hospital in the country; it has a 1,800-bed capacity and receives 89,000 admissions per year [13]. Mbagathi District Hospital is a smaller hospital that serves the local population with 200 beds and approximately 13,000 annual admissions [13]. The study participants were HIV-positive women between 19–49 years of age who were sexually active in the past 6 months and not currently pregnant. Participants had to have a CD4 count ≥ 350 cells/cc blood and be free of any AIDS-defining diseases (HIV stage I and II). Consent to the extraction of data from medical records was also required for participation. Five hundred and two eligible women were asked to participate in this study, 497 were enrolled and completed the survey (Figure 1). Twenty-one women had not disclosed their HIV status to anyone and 476 had disclosed. Of the 497 who participated, only 349 responded to our outcome question, “Who have you told about being HIV infected?” One-hundred and twenty seven observations were excluded due to not having a response to the question.

### Ethical approval

This research study was approved by the Institutional Review Board at the University of Alabama at Birmingham and the Kenyatta National Hospital/University of Nairobi Ethics and Research Committee.

### Participant recruitment

The clinic staff informed HIV-positive women who met the inclusion criteria about the study and asked if they were willing to participate. Women who expressed willingness to participate were then introduced to the research staff who conducted the informed consent process with the potential participants and administered a questionnaire to gather data on sociodemographic factors, social aid, stigma, social relationships, and use of Prevention of Mother-to-Child-Transmission (PMTCT) services, and reproductive autonomy after receiving signed informed consent. The variables are listed in tables 1–3. Clinical information such as HIV diagnosis date, CD4^+^ count, prior sexually transmitted infections (STIs), and viral load (if available) were obtained from the study participants’ medical records.

### Statistical analysis

The main outcome variable was the response to the question, “Who have you told about being HIV infected?” Those who disclosed to anyone other than their sexual partner (parent, child, friend, or religious leader) were merged to reflect individuals who disclosed their HIV-positive status to anyone other than a sexual partner. The sample size was calculated using the online EpiTool by Ausvet (http://epitools.ausvet.com.au) and a 65% HIV disclosure rate to sexual partner reported for Kenya by Farquhar C, et al [14]. The calculation estimated that 350 women were needed to assess our outcome with a 95% confidence level and 5% margin of error; our final sample of women who responded to our outcome variable was 349.

The sociodemographic, disclosure beliefs, health, and sexual behavior variables were categorized appropriately based on the responses to the questions on the survey and as shown in tables 1–3. For example, “Head of Household” (Self’, ‘Sexual partner/spouse’, ‘Father’, ‘Mother’, ‘Other’) and for “Final say in household about health care” (‘Me’, ‘Sexual partner/spouse’, ‘Father’, ‘Mother’, ‘Other’). No response was treated as missing. There were different numbers of missing responses across questions. Missing responses were removed just for the specific variable, changing some of the questions denominators. However, the report reflects the true proportion for that variable. Basic descriptive statistics were produced for all of the variables. Chi-square was run for categorical variables. In cases where the calculated values were <5 Fisher’s exact test was performed and t-test was run for continuous variables in order to identify differences among those who disclosed to their sexual partners *versus* those who disclosed to anyone other than their sexual partner. Following the bivariate analyses, a stepwise logistic regression model was conducted using the significant variables (p value <0.05) from the bivariate analysis. The logistic regression analysis was used to calculate the crude and adjusted odds of disclosure to a sexual partner compared to those who disclosed to someone other than a sexual partner using the stepwise indicated variables. Statistical analyses were performed using SAS (Statistical Analysis System, Cary, North Carolina, USA) 9.4 software, and all of the statistical tests were two-sided where α=0.05 was considered statistically significant.

## Results

Roughly, 54% of study participants had disclosed their HIV-positive status to a religious leader, 34% to a sexual partner, 7% to a family member who was not a spouse or sexual partner, and 2% to a friend (not shown). Results from table 1 show that the majority of women (66%) did not disclose to a sexual partner, and only 26% of those women who did not disclose were in a marital/common-law relationship (p<0.0001). Sixty-nine percent of women who had disclosed their HIV-positive status to someone other than a sexual partner reported themselves as the head of the household, compared to 46% of women who had disclosed to a sexual partner (p<0.0001). Also, disclosure to a sexual partner is higher if the sexual partner or spouse serves as the head of the household when compared to disclosure to someone other than a sexual partner (40% *vs*. 13%; respectively; p <0.0001).

Fifty-eight percent of women who disclosed their HIV-positive status to a sexual partner and 30% of those who disclosed to someone other than a sexual partner reported having a spouse <10 years older than them (p<0.0001; Table 1). Additionally, results showed that disclosure to a sexual partner is lower if the study participant reported themselves as the one who has the final say in the household about their healthcare (86% *vs*. 69%, respectively; p<0.0001). Among study participants, 71% of women who had disclosed their HIV-positive status to someone other than a sexual partner reported having zero pregnancies since their diagnosis, whereas only 48% of women who had disclosed to a sexual partner reported zero pregnancies (p<0.0001). Most participants who had disclosed their HIV-positive status to a sexual partner had two or more children alive (67%) and accessed PMTCT services during their last pregnancy (80%), compared to 53% and 63% of those who have disclosed their HIV-positive status to someone other than a sexual partner (p=0.0218 and p=0.0382). Lastly, there is a lower proportion of disclosure to a sexual partner if the study participant had <5 individuals living in their household (59% *vs*. 33%, respectively; p<0.0001).

Table 2 shows the difference between study participants’ HIV disclosure beliefs, well-being, and sexual behaviors. There was no significant difference between those who disclosed to a sexual partner and those who disclosed to someone other than a sexual partner; the sample of those who disclosed to a sexual partner had a CD4 count of approximately 574 (574.3 ± 216.1) and those who disclosed to someone other than a sexual partner had a CD4 count of approximately 582 (582.3 ± 246.0). While 80% of participants who had disclosed their HIV-positive status to a sexual partner felt secure in their relationship with their partner, only 60% of participants who disclosed to someone other than a sexual partner felt secure in their relationship (p=0.0009). Since their HIV diagnosis, 78% of those who had disclosed to a partner and 48% of those who disclosed to someone other than a sexual partner claim that their number of sexual partners had remained the same (p<0.0001). Additionally, results showed that disclosure to a sexual partner was higher if the study participant reported that the last time a condom was used stemmed from a joint decision with their partner (53% *vs*. 36%, respectively; p=0.0427). Disclosing one’s HIV-positive status typically occurred within 24 hours after the diagnosis for both those who had disclosed to a sexual partner (78%) and those who disclosed to someone other than a sexual partner (60%, p=0.0041). A majority of those who had disclosed to a sexual partner (65%) and those who had disclosed to someone other than a sexual partner (45%) disclosed their HIV-positive status because they felt it was the right thing to do (p=0.0177). There is also a higher proportion of disclosure to a sexual partner among women who report that they experienced abuse in their sexual relationship as an effect of their HIV-positive disclosure (79% *vs*. 61%, respectively, p=0.0252).

Furthermore, 88% and 86% of those who had disclosed to a sexual partner knew that there were medications to prevent MTCT of HIV during pregnancy and after birth compared to 75% of those who had disclosed to someone other than a sexual partner (p=0.0046 and p=0.0180). Regarding accessing reproductive health services and dual contraception, 94% of women who had disclosed to someone other than a sexual partner compared to 86% of women who had disclosed to a sexual partner reported that their partner did not accompany them during their visit (p=0.0304) and 90% of those who had disclosed to someone other than a sexual partner compared to 81% of those who had disclosed to a sexual partner did not practice condom use and an additional method of contraception (p=0.0328). Results show a higher proportion of women who disclosed their HIV-positive status to someone other than a sexual partner reported not using a condom since their HIV diagnosis (25% *vs*. 12%, respectively; p=0.0059). Additionally, 90% of women who disclosed to someone other than a sexual partner also reported believing that HIV disclosure is important for prevention and control compared to 99% of women who disclosed their HIV-positive status to a sexual partner (p=0.0010). Of those who had disclosed to a sexual partner, 85% knew their sexual partner’s HIV status compared to 64% of those who had not disclosed (p<0.0001).

The crude odds ratios and 95% Confidence Intervals (CI) for significant variables in the bivariate analyses are presented in table 3. Stepwise regression with a 0.1 threshold was utilized to determine the variables used in the adjusted model. Adjusted crude ratios are also presented in table 3. The odds of disclosing an HIV-positive status to a main sexual partner are 11 times more likely for women who are married or in a common-law relationship (OR=11.15, CI=6.52 to 19.09), 3 times more likely for women with five or more individuals living in the household (OR=2.97, CI= 1.87 to 4.72), and 2 times more likely for women who accessed PMTCT services during their last pregnancy (OR=2.42, CI=1.04 to 5.67). Feeling secure in one’s relationship (OR=2.71, CI=1.48 to 4.95), the sexual partner or spouse serving as the head of household (OR=3.69, CI=1.78 to 7.64), and partner have the final say in the woman’s healthcare (OR=5.24, CI=2.52 to 10.89) significantly increased the odds of disclosure to a sexual partner. Women whose number of sexual partners have remained the same since their diagnosis were 4 times more likely to disclose (OR=4.22, CI=2.46 to 7.23), and women who knew about taking medication to prevent MTCT of HIV during pregnancy and after birth were 2 times more likely to disclose their HIV-positive status to a sexual partner (OR=2.39, CI=1.27 to 4.53 and OR=2.0, CI=1.10 to 3.63). The odds of disclosing to a sexual partner was 3 times more likely for those who jointly decided with their sexual partner about condom use (OR=3.20, CI=1.27 to 8.03), 2 times more likely for those who have their partner accompany them to access reproductive health services (OR=2.31, CI=1.08 to 4.91), and 2 times more likely for those who practice condom use plus another method of contraception (OR=2.00, CI=1.06 to 3.77). Furthermore, the odds of disclosure were significantly increased among women who believed that HIV disclosure is important for HIV prevention and control (OR=12.57, CI=1.67 to 94.52) who knew of their sexual partner’s HIV status (OR=3.29, CI=1.76 to 6.13), and who reported using a condom 100% of the time (OR=2.84, CI=1.43 to 5.64). The odds of disclosing were 78% less likely for those who disclosed a few months after diagnosis but within a year after their diagnosis (OR=0.22 CI=0.08 to 0.60).

## Discussion

This study showed significant differences in sociodemographic, HIV disclosure beliefs, health, and sexual behavior factors among women living with HIV/AIDS who attended clinics at this Kenyatta National Hospital. These factors may influence participants to disclose or not disclose their HIV-positive status to a sexual partner; for instance, a majority of the women who reported being independent, not married, and having fewer persons in the household did not disclose their HIV-positive status to their sexual partners. Without social support from a significant other, women can be left to feel vulnerable to severe consequences of their HIV-positive status, which may include physical violence and abandonment [15]. Previous research among women found that despite the understood benefits of HIV-positive disclosure, the fear of disclosure and its perceived consequences outweighed the act of disclosing to a sexual partner [16]. This is especially true in low-income settings in Africa where the risk of intimate partner violence is increased for women living with HIV [17,18]. This study supports that finding as a majority of those who disclosed their HIV-positive status to a sexual partner were found to experience abuse as a result of disclosing. Therefore, in order to increase rates of HIV-positive disclosure among women living with HIV/AIDS, interventions that provide healthcare to HIV-positive women should include routine screening and intervention for domestic violence [19].

Conserve DF, et al. found that individuals who decided to disclose their status to a sexual partner were more likely to do so because of the risk of HIV transmission and the encouragement of their sexual partner to test for HIV [16]. This study found similar results as participants who knew their sexual partner’s HIV status were more likely to disclose their own HIV-positive status. Despite the fact that all participants in the study reported being sexually active, participants who had not disclosed may not have been in a stable or committed relationship. Participants who were married or in a common-law relationship were more likely to disclose their HIV-positive status to their sexual partner. Previous studies have shown similar results regarding married individuals or those living under common-law being more likely to disclose their HIV-positive status to sexual partners [20,21]. Marital/common-law status could impact disclosure as unmarried individuals often report negative reactions to disclosure that provoke violence and discrimination from their sexual partners [22]. This is consistent with our results that show women who felt more secure in their relationship were more likely to disclose their HIV-positive status to their sexual partner. Despite this finding, it is crucial for PLWHA to disclose to those with whom they engage in a sexual relationship in order to create safer relationships and prevent the transmission of HIV. Health care professionals should focus on educating individuals who have not disclosed their HIV-positive status to their sexual partners by demonstrating the importance of such disclosure.

Women who do not disclose their HIV-positive status to their sexual partners often engage in risky sexual practices such as inconsistent condom use [23]. Results from this study are concurrent with this evidence that participants who reported using a condom 100% of the time were more likely to have disclosed their HIV-positive status to a sexual partner. Bachanas P, et al. reported information consistent with the finding that individuals who disclosed their HIV-positive status were more likely to report using condoms consistently than those who had not disclosed [24]. The study shows that of the women who did not disclose to their sexual partners, a majority remained sexually active in addition to inconsistently using condoms, thereby increasing the risk for HIV. These women should disclose their HIV-positive status to their sexual partners, and if they do not want to disclose because of fear associated with the adverse effects on the relationship, interventions should be implemented to increase safe sexual practices during sexual encounters.

Since the study recruited participants from two high-level care hospitals in Kenya, a limitation of this study is limited generalizability of the sample that may not accurately indicate the prevalence of disclosure from women in rural settings, smaller clinics, or among those who do not seek treatment. Evidence shows that forced disclosure of HIV-positive status occurs at health care facilities due to being recognizable in health-care settings, while significant differences occur in disclosure practices between urban and rural settings [25]. Also, a primary limitation of the cross-sectional study design is the difficulty of establishing a temporal relationship between a response and an outcome; therefore, only an association can be inferred. Because a large number of predictors were included in the logistic regression model, a limitation could be the inclusion of too many predictor variables in the model caused by multicollinearity. Another limitation is that confirmation was not available when participants self-reported if they disclosed their HIV status to a sexual partner. Future research should focus on recruiting patients outside of hospital settings to attain accurate disclosure status prevalence throughout urban and rural areas of Kenya.

## Conclusion

HIV disclosure is important to prevent or reduce the risk of HIV transmission. An overall low prevalence of HIV-positive disclosure to a sexual partner and high prevalence of HIV-positive disclosure to someone other than a sexual partner among women living with HIV/AIDS in Kenyan hospital settings can be noted in this study. This study shows that women who are more socially independent are less likely to disclose, and those who have not disclosed to their sexual partner engage in risky sexual behaviors. These findings may help to create interventions that target factors associated with non-disclosure to sexual partners and lead to decrease in HIV transmission. Interventions should be focused on increasing the chances of disclosure to a sexual partner by educating individuals as to why HIV disclosure is important for HIV prevention and control. The importance of HIV disclosure regardless of marital status and independence concerning household and health care decisions should be emphasized to HIV-positive individuals. Targeting individuals who have not disclosed their HIV status to a sexual partner should be the primary focus for increasing HIV disclosure. Programs should additionally develop personalized, cost-effective interventions that focus on addressing such characteristics as risky sexual behaviors that demonstrate the importance of disclosure to those with whom they engage in a sexual relationship. Nevertheless, to make conclusions as to why some individuals have not disclosed their HIV status, further studies should be conducted among individuals in and out of hospital settings and within rural and urban populations.

## Figures and Tables

**Table 1:** Socio-demographic variables by HIV disclosure to sexual partner.

Variables	Sexual partner N=119 (34.3) N (%)	Other than sexual partner N=230 (65.7) N (%)	p-value
**Age**			**0.0033**
19–30	25 (21.0)	22 (9.6)	
31–40	65 (54.6)	123 (53.5)	
≥ 41	29 (24.4)	85 (37.0)	
**Marital status**			**<0.0001**
Married/Common Law	94 (79.7)	59 (26.0)	
Single/Divorced/Widow	24 (20.3)	168 (74.0)	
**Number of persons in household**			**<0.0001**
<5	39 (32.8)	136 (59.13)	
≥ 5	80 (67.2)	94 (41.2)	
**Head of household**			**<0.0001**
Self	55 (46.2)	158 (69.3)	
Sexual partner/spouse	47 (39.5)	30 (13.2)	
Father/Mother/Other	17(14.3)	40 (17.5)	
**How much old is your partner/spouse than you**			**<0.0001**
<10	68 (58.1)	66 (29.7)	
≥ 10	32 (27.4)	43 (19.6)	
Same age	17 (14.5)	113 (50.9)	
**Residential area**			0.1371
Rural	13 (11.1)	38 (17.2)	
Urban	104 (88.9)	183 (82.8)	
**Final say in household about your health care**			**<0.0001**
Me	81 (68.6)	198 (86.1)	
Sexual partner/spouse	26 (22.0)	12 (5.2)	
Father/Mother/Other	11 (9.3)	20 (8.7)	
**Number of children alive**			**0.0218**
0	10 (8.5)	37 (16.4)	
1	29 (24.6)	70 (29.9)	
≥ 2	79 (66.9)	119 (52.7)	
**How many pregnancies have you had since your diagnosis?**			**<0.0001**
0	57 (47.9)	159 (70.9)	
1	46 (38.7)	55 (24.6)	
≥ 2	16 (13.4)	10 (4.5)	
**Did you access PMTCT services during your last pregnancy?**			**0.0382**
Yes	49 (80.3)	32 (62.8)	
No	12 (19.7)	19 (37.2)	
**Social status**			0.0565
High	5 (4.2)	3 (1.3)	
Average	79 (67.0)	137 (59.8)	
Low/Below poverty line	34 (28.8)	89 (38.9)	
**Education**			0.1177
Primary	23 (19.3)	65 (28.3)	
Secondary	54 (45.4)	96 (41.7)	
Tertiary	41 (34.5)	62 (27.0)	
None	1 (0.8)	7 (3.0)	
**Employment status**			0.1649
Employed/Self-employed	78 (66.7)	169 (73.8)	
Unemployed	39 (33.3)	60 (26.2)	
**Religion**			0.9639
Christian	116 (98.3)	223 (98.2)	
Other/Muslim/None	2 (1.7)	4 (1.8)	

* Numbers may not add to total number of participants due to missing responses; Bold=significant at p<0.05

**Table 2:** HIV disclosure beliefs and health and sexual behavior by HIV disclosure to sexual partner.

Variables	Sexual partner N=119 (34.3) N (%)	Other than sexual partner N=230 (65.7) N (%)	p-value
CD4 count (mean ± standard deviation)	574.3 ± 216.1	582.3 ± 246.0	0.7654
**Do you feel secure in your relationship with your partner?**			**0.0009**
Yes	86 (80.4)	71 (60.2)	
No	21 (19.6)	47 (39.8)	
**Are you a part of a social support group?**			**0.0213**
Yes	13 (10.9)	47 (20.4)	
No	106 (89.1)	183 (79.6)	
**Since your HIV diagnosis, your number of sexual partners have**			**<0.0001**
Remained the same	87 (77.7)	105 (46.7)	
Increased	3 (2.7)	8 (3.6)	
Decreased	22 (19.6)	112 (49.8)	
**If you used a condom the last time you had sex, who initiated the condom use?**			**0.0427**
I did	16 (28.6)	19 (52.8)	
My partner	5 (8.9)	4 (11.1)	
Joint decision	35 (62.5)	13 (36.1)	
**How soon after diagnosis did you disclose your HIV status to someone?**			**0.0041**
<24 Hours	93 (78.2)	123 (60.0)	
Days	7 (5.9)	13 (6.3)	
Weeks	5 (4.2)	13 (6.3)	
≤ 12 Months	5 (4.2)	31 (15.2)	
≥ 1 Year	9 (7.5)	25 (12.2)	
**What was the main reason for disclosing your HIV status?**			**0.0177**
You felt it was the right thing to do	60 (65.2)	75 (44.7)	
Fear	4 (4.4)	12 (7.1)	
You were getting sick	24 (26.1)	69 (41.1)	
You wanted access to services	4 (4.4)	12 (7.1)	
**What has been the effect of your HIV disclosure on your sexual relationship?**			**0.0252**
Strengthened relationship	8 (8.2)	15 (15.5)	
Resulted in separation	13 (13.2)	23 (23.7)	
Experienced abuse	77 (78.6)	59 (60.8)	
**Do you know about taking medication to prevent transmission of HIV to your baby during pregnancy?**			**0.0046**
Yes	103 (88.0)	166 (75.5)	
No	14 (12.0)	54 (24.5)	
**Do you know about taking medication to prevent transmission of HIV after the birth of your baby?**			**0.018**
Yes	102 (85.7)	168 (75)	
No	17 (14.3)	56 (25)	
**Does your partner accompany you to access reproductive health services?**			**0.0304**
Yes	16 (13.6)	14 (6.4)	
No	102 (86.4)	206 (93.6)	
**Do you practice dual protection (condom use + another method of contraception?**			**0.0328**
Yes	22 (18.6)	23 (10.3)	
No	96 (81.4)	201 (89.7)	
**When were you diagnosed with HIV**			0.8057
<1 year	10 (8.4)	15 (6.5)	
1–5 years	65 (54.6)	121 (52.6)	
6–10 years	34 (28.6)	69 (30.0)	
>10 years	10 (8.4)	25 (10.9)	
**Health care provider told me to disclose my HIV status**			0.5624
Yes	69 (93.2)	68 (90.4)	
No	5 (6.8)	7 (9.3)	
**Health care provider explained ways I could disclose my HIV status**			0.1769
Yes	40 (93.0)	132 (85.7)	
No	3 (7.0)	22 (14.3)	
**Do you believe HIV disclosure is important for HIV prevention and control**			**0.0010**
Yes	112 (99.1)	196 (89.9)	
No	1 (0.9)	22 (10.1)	
**Do you think the benefit of HIV disclosure outweigh the risk**			0.4211
Yes	88 (79.3)	165 (75.1)	
No	23 (20.7)	54 (24.9)	
**Did you use a condom the last time you had sex**			0.0891
Yes	92 (83.6)	134 (75.2)	
No	18 (16.4)	44 (24.7)	
**Frequency of condom use since diagnosis**			**0.0059**
Do not use condom	13 (11.8)	45 (25.4)	
Use condom some/most times	20 (18.2)	38 (21.5)	
Use condom 100% of the time	77 (70.0)	94 (53.1)	
**Do you know the HIV status of your partner**			**<0.0001**
Yes	92 (85.2)	98 (63.6)	
No	16 (14.8)	56 (36.4)	

* Numbers may not add to total number of participants due to missing responses; Bold=significant at p<0.05

**Table 3:** Odds of disclosure of HIV status to their main sexual partner among women who are HIV-positive.

Variables	Crude OR (95% CI)	Adjusted OR (95% CI)
**Age**		
19–30	Ref	
31–40	**0.47 (0.24 to 0.89)**	
≥ 41	**0.30 (0.15 to 0.61)**	
**Number of persons in household**		
<5	Ref	
≥ 5	**2.97 (1.87 to 4.72)**	
**Marital status**		
Married/Common Law	**11.15 (6.52 to 19.09)**	
Single/Divorced/Widow	Ref	
**Number of children alive**		
0	0.64 (0.28 to 1.44)	0.615 (0.26 to 1.43)
1	Ref	Ref
≥ 2	1.59 (0.95 to 2.67)	1.50 (0.88 to 2.58)
**Did you access PMTCT services during your last pregnancy?**		
Yes	**2.42 (1.04 to 5.67)**	
No	Ref	
**How much old is your partner/spouse than you?**		
<10	1.38 (0.78 to 2.45)	
≥ 10	Ref	
Same age	**0.20 (0.10 to 0.40)**	
**Final say in household about your health care**		
Sexual partner/spouse	**5.24 (2.52 to 10.89)**	
Parents	0.92 (0.24 to 3.54)	
Me	Ref	
**Head of household**		
Self	0.82 (0.43 to 1.56)	
Sexual partner/spouse	**3.69 (1.78 to 7.64)**	
Father/Mother/Other	Ref	
**Do you feel secure in your relationship with your partner?**		
Yes	**2.71 (1.48 to 4.95)**	
No	Ref	
**Are you a part of a social support group?**		
Yes	**0.48 (0.25 to 0.92)**	
No	Ref	
**Since your HIV diagnosis, your number of sexual partners have**		
Remained the same	**4.22 (2.46 to 7.23)**	
Increased	1.91 (0.47 to 7.77)	
Decreased	Ref	
**Do you know about taking medication to prevent transmission of HIV to your baby during pregnancy?**		
Yes	**2.39 (1.27 to 4.53)**	
No	Ref	
**Do you know about taking medication to prevent transmission of HIV after the birth of your baby?**		
Yes	**2.00 (1.10 to 3.63)**	
No	Ref	
**If you used a condom the last time you had sex, who initiated the condom use?**		
I did	Ref	
My partner	1.48 (0.34 to 6.48)	
Joint decision	**3.20 (1.27 to 8.03)**	
**How soon after diagnosis did you disclose your HIV status to someone?**		
<24 Hours	Ref	Ref
Days	0.71 (0.27 to 1.86)	0.69 (0.26 to 1.82)
Weeks	0.51 (0.18 to 1.48)	0.55 (0.19 to 1.62)
≤ 12 Months	**0.21 (0.08 to 0.57)**	**0.22 (0.08 to 0.60)**
≥ 1 Year	0.48 (0.21 to 1.07)	0.50 (0.22 to 1.13)
**Does your partner accompany you to access reproductive health services?**		
Yes	**2.31 (1.08 to 4.91)**	
No	Ref	
**Do you practice dual protection (condom use+another method of contraception?**		
Yes	**2.00 (1.06 to 3.77)**	
No	Ref	
**Do you believe HIV disclosure is important for HIV prevention and control**		
Yes	**12.57 (1.67 to 94.52)**	
No	Ref	
**Knowledge of HIV status of sexual partner**		
Yes	**3.29 (1.76 to 6.13)**	
No	Ref	
**Frequency of condom use since diagnosis**		
Do not use condom	Ref	
Use condom some/most times or 100% of time	1.82 (0.80 to 4.14)	
Use condom 100% of the time	**2.84 (1.43 to 5.64)**	

* Bold=significant at p<0.05
